# Evolution of Microstructural and Mechanical Properties during Cold-Rolling Deformation of a Biocompatible Ti-Nb-Zr-Ta Alloy

**DOI:** 10.3390/ma15103580

**Published:** 2022-05-17

**Authors:** Alexandru Dan, Mariana Lucia Angelescu, Nicolae Serban, Elisabeta Mirela Cojocaru, Nicoleta Zarnescu-Ivan, Vasile Danut Cojocaru, Bogdan Mihai Galbinasu

**Affiliations:** 1 Faculty of Materials Science and Engineering, University Politehnica of Bucharest, 060042 Bucharest, Romania; alexandru.dan0806@upb.ro (A.D.); mariana.angelescu@upb.ro (M.L.A.); nicolae.serban@upb.ro (N.S.); elisabeta.cojocaru@upb.ro (E.M.C.); nicoleta.zarnescu@upb.ro (N.Z.-I.); 2 Dental Medicine Faculty, University of Medicine and Pharmacy “Carol Davila” Bucharest, 020021 Bucharest, Romania; bogdan.galbinasu@umfcd.ro

**Keywords:** Ti-biocompatible alloys, SEM and XRD microstructural analysis, mechanical testing

## Abstract

In this study, a Ti-32.9Nb-4.2Zr-7.5Ta (wt%) titanium alloy was produced by melting in a cold crucible induction in a levitation furnace, and then deforming by cold rolling, with progressive deformation degrees (thickness reduction), from 15% to 60%, in 15% increments. The microstructural characteristics of the specimens in as-received and cold-rolled conditions were determined by XRD and SEM microscopy, while the mechanical characteristics were obtained by tensile and microhardness testing. It was concluded that, in all cases, the Ti-32.9Nb-4.2Zr-7.5Ta (wt%) showed a bimodal microstructure consisting of Ti-β and Ti-α″ phases. Cold deformation induced significant changes in the microstructural and the mechanical properties, leading to grain-refinement, crystalline cell distortions and variations in the weight-fraction ratio of both Ti-β and Ti-α″ phases, as the applied degree of deformation increased from 15% to 60%. Changes in the mechanical properties were also observed: the strength properties (ultimate tensile strength, yield strength and microhardness) increased, while the ductility properties (fracture strain and elastic modulus) decreased, as a result of variations in the weight-fraction ratio, the crystallite size and the strain hardening induced by the progressive cold deformation in the Ti-β and Ti-α″ phases.

## 1. Introduction

Over time, titanium and titanium alloys have proven to be the most desirable alloys for medical applications, such as orthopaedic and dental implants, due to their excellent biocompatibility, low elastic modulus, high strength-to-weight ratio and corrosion resistance [1,2,3,4,5,6]. Due to the stiffness of the classic Ti-6Al-4V alloy [7,8,9] and due to the toxicity of Al and V [10,11], alternatives to this alloy, which may offer either the same or even improved mechanical and biocompatibility properties, have been continually sought. The elements that provide these properties in combination with Ti are Nb, Zr, Mo and Ta, which are known to be β stabilizers, and were found to be the most biocompatible chemical elements used in metallic implants, causing minimal adverse side effects in the human body [12,13,14,15,16]. For this reason, a new generation of β-type Ti alloys has been developed: alloys that have a low elastic modulus, similar to that of human bones, containing Nb, Ta and Zr [17,18,19,20,21,22,23,24,25].

Among the Ti alloys without Al and V, the alloys belonging to the Ti-Nb-Zr-Ta (TNZT) system are some of the most promising alloys for orthopaedic implants [26,27,28,29,30,31,32,33]. After cold deformation, at room temperature, TNZT alloys exhibit excellent properties: low elastic modulus, high yield strength, superelasticity, superplasticity and good corrosion resistance, all of which make them excellent candidates for implant materials. The demand of a low elastic modulus, as close as possible to the value of 30 GPa (the maximum elastic modulus of human bone is 15–30 GPa), is important in order to avoid the occurrence of the stress-shielding effect, which leads to bone atrophy [2,3,4,5,6] and represents one of the biggest challenges in orthopaedic implant design [34,35,36,37,38,39]. 

It was also reported that metastable β-type Ti-alloys have a better cold formability and a lower elastic modulus than α and near-α or α + β Ti alloys [40,41,42,43]; therefore, the development of new β-type Ti alloys containing non-toxic elements such as Nb, Zr and Ta is very important. Both chemical composition and microstructure have an important influence on the biocompatibility of Ti-based alloys. It has been proven that the addition of Nb to Ti-based alloys improves their corrosion resistance to saline solutions, that the addition of Ta provides good corrosion resistance and superior mechanical properties, and that the addition of Zr provides solid solution strengthening [44,45,46,47,48,49]. Establishing a correlation between composition, microstructure and mechanical properties in Ti alloys is necessary. By tailoring the thermomechanical processing route, mechanical characteristics, such as ductility and toughness, can increase significantly [38,39]. Depending on the alloy’s composition, different mechanisms such as dislocation slip, twinning or stress-induced martensite (SIM), or a combination of them, can occur in order to accommodate the applied stress [40,41,42].

Based on these reasons, the present work aims to study the correlation between conventional processing by cold plastic deformation and the microstructural and mechanical properties of a biocompatible TNZT alloy, in order to obtain the optimum possible combination of strength and ductility properties necessary for biomedical applications.

## 2. Materials and Methods

### 2.1. Alloy Manufacturing and Its Processing Route

**Figure 1:**
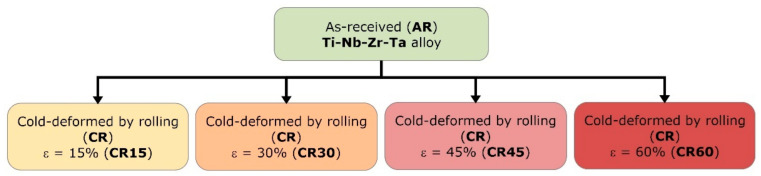
Processing scheme applied to Ti-32.9Nb-4.2Zr-7.5Ta (wt.%) alloy.

The investigated Ti-32.9Nb-4.2Zr-7.5Ta (wt%) (TNZT) alloy was obtained by melting in an inert controlled atmosphere (argon) in a cold crucible (in levitation) FIVE CELES-MP25 (Five’s Group Company, Paris, France) furnace, starting from high-purity elemental components: Ti: min. 99.6%, no. GF71176776; Nb: min. 99.9%, no. GF49338120; Zr: min. 99.5%, no. GF10742284; Ta: min. 99.9%, no. GF80066392 (SIGMA ALDRICH/MERCH, Merck KGaA, Darmstadt, Germany). In order to obtain a high chemical homogeneity, the ingots were re-melted twice. The alloy’s chemical composition was determined by the EDS technique, using a scanning electron microscope (SEM), a TESCAN VEGA II—XMU model (TESCAN, Brno, Czech Republic), coupled with a BRUKER Quantax xFlash 6/30 (Bruker Corporation, Billerica, MA, USA) EDS detector. Figure 1 shows the processing path applied to the TNZT alloy, starting from the initial/as-received (AR) state, to the cold-rolled states (CR), with increasing total deformation degrees (thickness reductions) from approx. ε ≈ 15% to ε ≈ 60%, in four steps with ε ≈ 15%/step, using a Mario di Maio LQR120AS rolling-mill (Mario di Maio Inc., Milano, Italy).

### 2.2. Microstructural and Mechanical Characterization

The microstructural investigations were performed using an XRD table-top RIGAKU MiniFlex600 (RIGAKU, Tokyo, Japan) diffractometer, using Cu radiation (Cu-Kα; λ~1.54Å) and a TESCAN VEGA II–XMU (TESCAN, Brno, Czech Republic) SEM microscope. The polishing and super-polishing procedure steps are presented in detail in a previous paper [50]. To highlight the microstructure of the TNZT alloy, after the super-polishing step, the samples were etched with an etching solution (Kroll reagent) with the following composition: 6 mL nitric acid (HNO_3_) + 3 mL hydrofluoric acid (HF) + 91 mL distilled water, the etching duration being 40 to 60 s.

Mechanical characterization by tensile tests was performed using a GATAN Micro Test-2000N testing machine (Gatan Inc., Pleasanton, CA, USA), with a strain rate of 1 × 10^−4^ s^−1^. The tensile testing procedure is presented in detail in a previous paper [51]. Mechanical characterization by micro-hardness testing was performed using an INNOVATEST Falcon 500 (INNOVATEST Europe BV, Maastricht, Netherlands) equipment with a 200 gf testing force and a 30 s dwell time. The mechanical testing procedure is presented in detail in a previous paper [52]. The following mechanical characteristics were measured: ultimate tensile strength (σ_UTS_), yield strength (σ_0.2_), fracture strain (ε_f_), elastic modulus (E) and microhardness (HV0.2). All values were rounded to the nearest integer.

## 3. Results and Discussion

### 3.1. The β-Phase Stability Analysis of Our TNZT Alloy

Some authors have divided Ti alloys into three main classes [53,54,55,56], such as: α and near-α ([Mo]eq. < 2), α + β ([Mo]eq. = 2–5), near-β ([Mo]eq. = 5–10), β-metastable ([Mo]eq. = 10–30) and stable β ([Mo]eq. > 30) alloys, based on the alloying elements content. Alloying elements can be either α or β stabilizers, and, as such, can increase or decrease the phase proportions in Ti-based alloys. The stability of the β phase is defined by chemical composition and can be established by using the Molybdenum equivalency, considering that Mo is a typical β-forming element. The Mo equivalency ([Mo]eq., wt%) can be deduced from the quantities of β-stabilizing elements by using the following equation [55,56]:

(1) $${\left[\mathrm{Mo}\right]}_{\mathrm{eq}.}=1\cdot \mathrm{Mo}+0.28\cdot \mathrm{Nb}+0.22\cdot \mathrm{Ta}+0.67\cdot V+1.6\cdot \mathrm{Cr}+2.90\cdot \mathrm{Fe}-{\left[\mathrm{Al}\right]}_{\mathrm{eq}.}$$

(2) $${\left[\mathrm{Al}\right]}_{\mathrm{eq}.}=1\cdot \mathrm{Al}+0.17\cdot \mathrm{Zr}+0.33\cdot \mathrm{Sn}+10\left(O+N\right)$$

In this study, taking into account the determined alloy’s chemical composition (Table 1), the calculated value of [Mo]eq. was [Mo]eq. = 10.14, which classifies the TNTZ alloy as β-metastable ([Mo]eq. > 10) and shows that it can be transformed to martensite by a temperature- and/or stress-induced mechanism.

### 3.2. Microstructural Evolution during Cold Rolling

#### 3.2.1. SEM-EDS Microstructural Analysis

**Figure 2:**
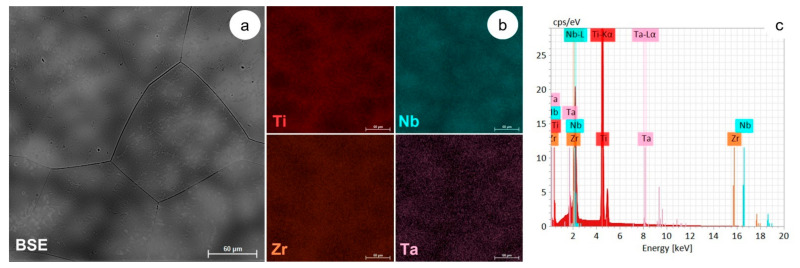
SEM-BSE image of the AR TNZT alloys microstructure (**a**); SEM-EDS colorized maps showing the main alloying elements distribution (**b**); EDS spectra of AR TNZT alloy (**c**).

The microstructural analysis of the TNTZ alloy used in this study was performed using the SEM-EDS technique. A representative SEM image of the as-received (AR) TNZT alloy obtained with a BSE (backscattered electron detector) is shown in Figure 2a, where one can observe that the microstructure consists of polyhedral Ti-β-phase grains, with an average grain size above 150 μm, which contains the submicron-size Ti-α″ phase. Figure 2b illustrates the distribution of the main alloying elements (Ti, Nb, Zr and Ta) obtained with the EDS elemental map. In Figure 2c, the obtained EDS spectra is illustrated. According to the EDS map and spectra, the TNZT alloy presents a good chemical homogeneity, with no other alloying elements being found in its chemical composition.

The samples used in this study were subjected to deformation by cold rolling, with various deformation degrees, gradually increasing from 15% to 60%, in 15% steps. All the cold-rolled samples were studied in the RD–ND cross-section (RD—rolling direction; ND—normal direction), but only a few representative images were selected in order to highlight the microstructural characteristics.

**Figure 3:**
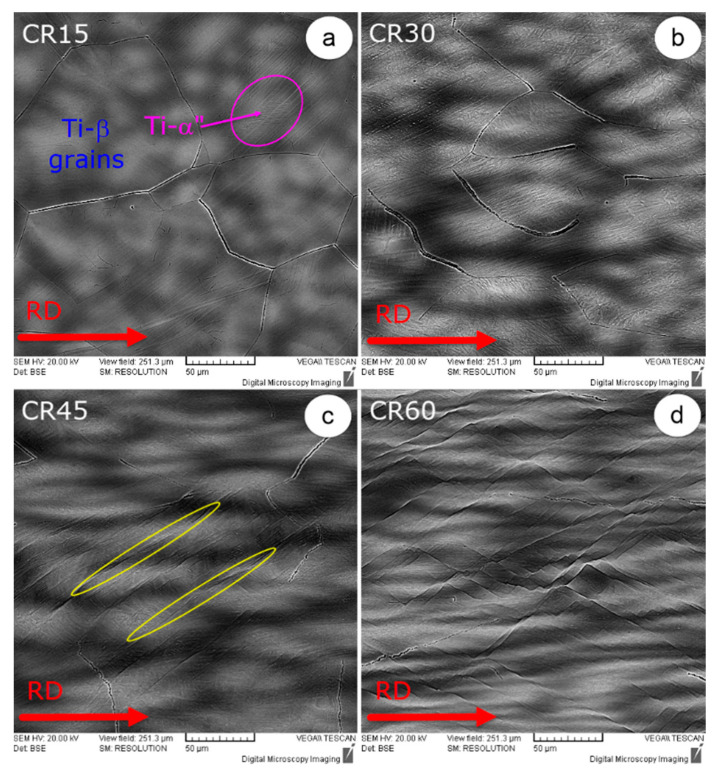
Microstructure evolution during cold deformation processing: 15% cold-rolled (CR15) (**a**); 30% cold-rolled (CR30) (**b**); 45% cold-rolled (CR45) (**c**); 60% cold-rolled (CR60) (**d**).

Figure 3 shows the SEM images of the cold-rolled TNZT studied samples. It can be seen that in the rolling direction, the initial polyhedral Ti-β-phase grains become more and more elongated as the degree of deformation increases.

The Ti-α″ phase shows an acicular/plate-like morphology being contained within the parent Ti-β-phase grains. It can be also remarked that deformation bands and twins become more and more visible as the intensity of cold-rolling deformation increases. Similar observations were obtained in the case of different types of Ti alloys [57,58,59,60,61,62,63,64,65,66]. The most obvious microstructural features induced by the intense deformation are shown in Figure 3d, which illustrates the microstructure of the TNZT alloy when subjected to a 60% deformation degree.

#### 3.2.2. XRD Microstructural Analysis

**Figure 4:**
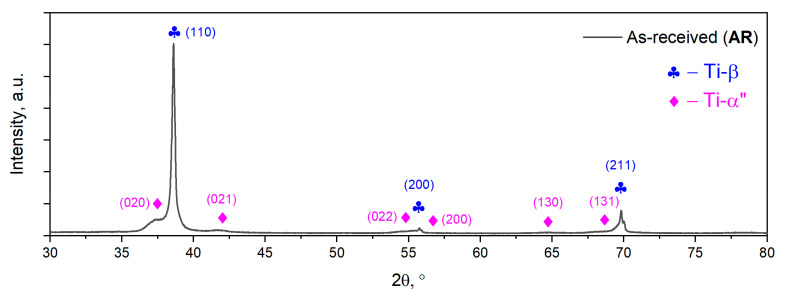
XRD spectra of the AR TNZT alloy.

The microstructural characterization of the AR TNZT alloy using the XRD technique aims to analyze the microstructure phase constituents. The obtained XRD spectra of the AR TNZT alloy is presented in Figure 4. One can observe that the AR TNTZ alloy microstructure consists of two phases, namely Ti-β and Ti-α″. The Ti-β phase is pointed out by (110), (200) and (211) diffraction lines, while the Ti-α″ phase is pointed out by (020), (021), (022), (200), (130) and (131) diffraction lines.

The Ti-β phase was indexed in the body-centered cubic (BCC) system, belonging to the *229/I m −3 m* space group, with a lattice parameter close to *a*_β_ = 0.329 nm, while the Ti-α″ martensite phase was indexed in the orthorhombic (O) system, belonging to the *63/C m c m* space group, with the lattice parameters *a*_α″_  =  0.298 nm, *b*_α″_  =  0.492 nm and *c*_α″_  =  0.463 nm. 

Considering the crystallographic fit between the parent BCC and transformed O crystalline cell, one can notice a misfit between the expected and the observed lattice parameters of the transformed Ti-α″ phase. For a “perfect” fit between the parent Ti-β phase, with a lattice parameter of *a*_β_ = 0.329 nm, and the transformed Ti-α″ phase, the Ti-α″ phase should possess lattice parameters close to *a*_α″_ = 0.288 nm, *b*_α″_ = 0.526 nm and c_α″_ = 0.473 nm [67,68]. The observed misfit shows an increase in the lattice parameter *a*_α″_ from the expected value of 0.288 nm to the observed value of 0.298 nm, and a decrease in both b_α″_ and c_α″_ lattice parameters from 0.526 nm to 0.492 nm and 0.473 nm to 0.463 nm, respectively. These kinds of differences are observed in many other titanium-based alloys containing Nb [69], Zr [70,71], Ta [72,73], V [74,75] and Mo [67,74], all of which show a continuous variation of the network parameters with increasing concentration of dissolved elements.

The X-ray diffraction pattern of AR TNZT alloy shows much stronger diffraction lines in the case of the Ti-β phase compared with the Ti-α″ martensite phase, suggesting that the weight fraction of the transformed Ti-α″ martensite phase is much lower compared with the weight fraction of the parent Ti-β phase (see Figure 4)

**Figure 5:**
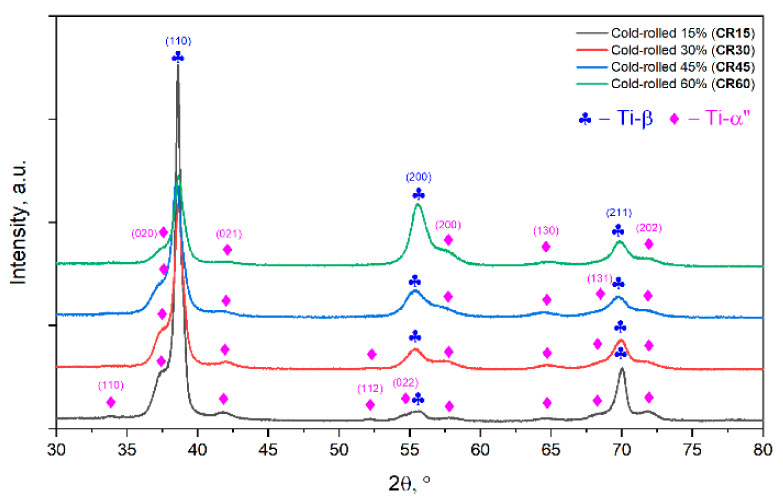
XRD spectra of the cold-rolled (CR) TNZT alloy.

Figure 5 shows the XRD spectra of the cold-rolled specimens (CR) with a total deformation degree (thickness reduction) of 15% (CR15), 30% (CR30), 45% (CR45) and 60% (CR60), respectively. The following qualitative observations can be highlighted.

In all cases, only diffraction lines belonging to the Ti-β and Ti-α” phases were detected; the X-ray diffraction patterns of cold-rolled specimens show stronger diffraction lines in the case of the Ti-β phase compared with the Ti-α″ phase, but with a lower-intensity ratio Imax_β_/Imax_α″_ compared with the initial AR state, suggesting that the weight fraction of transformed Ti-α″ phase is higher in all CR states. Additionally, when comparing the CR states, it can be seen that increasing the applied degree of deformation from 15% to 60% leads to a rise in the weight fraction of the transformed Ti-α″ phase, suggesting that the Ti-β → Ti-α″ phase transformation is highly stress/strain-dependent.

Analyzing the broadening evolution of the diffraction lines during cold rolling, one can notice an increase in the broadening of diffraction lines of both Ti-β and Ti-α″ phases, which suggests a noticeable reduction in the average crystallite size of both Ti-β and Ti-α″ phases, proving that cold rolling induces an intense grain refining of both Ti-β- and Ti-α″-phase grains. This assumption is based on the Scherrer formalism, which gives a correspondence between the diffraction line broadening (*FWHM* parameter) and the average coherent crystallite size [76]:

(3) $$D=\left(k\;\times \;\lambda \right)/\;\left(FWHM\times \cos\theta \right)$$

where *D*—average crystallite size, k—shape factor, λ—wavelength of the XRD radiation, *FWHM*—full width at half-maximum/broadening of the considered diffraction line and θ—diffraction line position.

Analyzing the Scherrer equation, it can be seen that the diffraction line broadening (*FWHM* parameter) and the average crystallite size are inversely proportional, with the crystallite size (*D*) decreasing with the increase in diffraction line broadening (*FWHM*).

Besides changes in diffraction line broadening, one can notice changes in the diffraction line intensity, especially in the case of the Ti-β phase, namely the initial intensity ratios I_β(110)_/I_β(200)_, I_β(110)_/I_β(211)_ and I_β(200)_/I_β(211)_ undergo changes while increasing the applied deformation degree, suggesting that a deformation texture (preferential crystalline orientation) is developing with the progress of deformation.

Analyzing the changes in diffraction lines position for both Ti-β and Ti-α″ phases, a slight shift towards higher 2θ values can be noticed, suggesting the occurrence of a crystalline cell’s compression in both Ti-β and Ti-α″ phases. This assumption is based on Bragg’s law, which gives a correspondence between the diffraction line position and the crystalline cell lattice spacing [77]:

(4) n×λ=2d×sinθ

where *n*—diffraction order, *λ*—wavelength of the XRD radiation, d—lattice spacing/interplanar distance and θ—diffraction line position. 

Analyzing Bragg’s law, it can be seen that the diffraction line position and the lattice spacing are inversely proportional, the interplanar distance (d) decreasing with the increase in the diffraction line position (θ).

### 3.3. Mechanical Properties’ Evolution during Cold Rolling

In order to study the influence of cold-rolling deformation on the mechanical properties of the TNZT alloy, all tests were performed on both as-received (AR) samples and cold-rolled (CR) state samples of the TNZT alloy. Based on the engineering strain—stress curves, the values of the main mechanical properties measured in this research work—ultimate tensile strength (σ_UTS_), yield strength (σ_0.2_), fracture strain (ε_f_), elastic modulus € and microhardness (HV0.2), respectively, are summarized in Table 2. 

The observed mechanical properties’ evolution from the AR initial state to the 15% cold-deformed (CR15) state (increase in the ultimate tensile strength from 708 ± 10 MPa to 963 ± 14 MPa, increase in the yield strength from 512 ± 13 MPa to 844 ± 13 MPa, decrease in the fracture strain from 10% ± 2% to 8% ± 1%, decrease in the elastic modulus from 59 ± 2 GPa to 57 ± 2 GPa and increase in the microhardness from 219 ± 4 HV0.2 to 244 ± 7 HV0.2) show that for all phenomena, the increase in the weight fraction of the stress-induced Ti-α″ phase, the grain-refinement and the occurrence of strain-hardening in both Ti-β and Ti-α″ phases, are significant and must be considered.

The analysis of mechanical characteristics/properties evolution with the progress of applied deformation degree, from 15% (CR15) to 60% (CR), show similar trends: increase in the ultimate tensile strength from 963 ± 14 MPa to 1261 ± 12 MPa, increase in the yield strength from 844 ± 13 MPa to 1166 ± 10 MPa, decrease in the fracture strain from 8% ± 1% to 4% ± 1 %, decrease in the elastic modulus from 57 ± 2 GPa to 50 ± 3 GPa and increase in the microhardness from 244 ± 7 HV0.2 to 257 ± 11 HV0.2. Additionally, these observations show that, for all phenomena, the increase in the weight fraction of the stress-induced Ti-α″ phase, grain-refinement and the occurrence of strain-hardening in both Ti-β and Ti-α″ phases, are significant and must be taken into account. However, considering the specifics of the microstructural changes occurring during plastic deformation, it is likely that the grain-refinement and strain-hardening phenomena play the leading role in influencing the mechanical behavior.

In order to explain the changes in the mechanical properties which result from cold-deformation processing, one must take into account the changes in the alloy’s microstructure by considering the following: the law of phase mixture, the grain-size evolution and the strain-hardening phenomena. The law of phase mixture states that the higher the weight fraction of a phase, the higher the influence of this phase on the alloy’s overall mechanical behavior [78,79]. Additionally, one must consider how the influence of the size of crystalline grains on mechanical properties, per the Hall–Petch correlation [80,81,82], and the strain-hardening phenomena, occurring due to the increased density of defects at crystalline level, lead to an increase in the strength properties and a decrease in the ductility [83,84,85]. 

As observed from XRD analysis (see Figure 5), even at a 15% applied deformation degree, one can notice the signs of the increased weight fraction of the stress-induced Ti-α″ phase, which can lead to a decrease in both strength properties and elastic modulus, with it being known that the Ti-α″ phase exhibits a much lower strength and elastic modulus in comparison with the parent Ti-β phase [81,82]. It was shown that, even for small applied plastic strains, the weight fraction of the stress-induced transformed Ti-α″ phase was considerable [81,82] due to the relatively low triggering stress for stress-induced martensitic transformation Ti-β → Ti-α”. The observed crystallite size evolution shows the grain refinement is present and leads to a reduction in size with the progress of the applied degree of deformation, and, as a consequence of the Hall–Petch correlation, to increased strength properties. Additionally, signs of induced strain hardening are visible, leading to an increase in the necessary stress needed to accommodate the applied strain and, therefore, to an increase in the strength properties and a decrease in ductility [83,84,85]. 

## 4. Conclusions

A β-metastable Ti-32.9Nb-4.2Zr-7.5Ta (wt%) (TNZT) alloy was fabricated by melting in a cold crucible induction in a levitation furnace and subjected to cold deformation, by rolling, with progressive deformation degrees, from 15% to 60%, in 15% increments, in order to investigate the microstructural changes and the obtained mechanical properties. 

The obtained results can be summarized as follows:

- The investigated β-metastable Ti-32.9Nb-4.2Zr-7.5Ta (wt%) alloy can support the Ti-β → Ti-α″ martensite phase transition;
- The microstructure of the as-received (AR) Ti-32.9Nb-4.2Zr-7.5Ta (wt%) alloy consists of a dual-phase microstructure, composed of polyhedral Ti-β-phase grains containing a submicron-sized dispersed Ti-α″ phase;
- Increasing the applied deformation degree leads to changes in the morphology of both Ti-β and Ti-α″ phases, leading to the development of elongated Ti-β-phase grains containing the transformed acicular/plate-like Ti-α″ phase;
- Increasing the applied deformation degree leads to crystalline cell compression, decreased crystallite size and changes in the weight fraction of both Ti-β and Ti-α″ phases, influencing the Ti-32.9Nb-4.2Zr-7.5Ta (wt%) alloy’s exhibited mechanical behavior by increasing the ultimate tensile strength, yield strength and microhardness, and decreasing the elastic modulus and fracture strain;
- An excellent combination of mechanical properties, high strength (>1200 MPa) and low elastic modulus (<50 GPa), can be obtained by applying a cold deformation with a total applied deformation degree of 60%.

## Figures and Tables

**Table 1:** The quantitative chemical composition of the as-received (AR) TNZT alloy.

Element	At. No.	Mass, [% wt.]	Mass, [% at.]	Abs. Error, [%]	Rel. Error, [%]
Titanium	22	55.38	72.37	1.87	2.79
Niobium	41	32.89	22.14	1.07	3.74
Zirconium	40	4.22	2.89	0.27	4.16
Tantalum	73	7.51	2.60	0.13	3.31
Sum		100.00	100.00		-

**Table 2:** Mechanical characteristics of AR and CR TNZT alloy.

Structural State	Ultimate Strength, σ_UTS_ [MPa]	Yield Strength, σ_0.2_ [MPa]	Fracture Strain, ε_f_ [%]	Elastic Modulus, E [GPa]	Microhardness, HV0.2
As-received (AR) TNZT	708 ± 10	512 ± 13	10 ± 2	59 ± 2	219 ± 4
Cold-rolled 15% (CR15)	963 ± 14	844 ± 13	8 ± 1	57 ± 2	244 ± 7
Cold-rolled 30% (CR30)	109 ± 15	951 ± 11	6 ± 1	55 ± 2	248 ± 6
Cold-rolled 45% (CR45)	1133 ± 13	1011 ± 12	5 ± 1	52 ± 3	245 ± 8
Cold-rolled 60% (CR60)	1261 ± 12	1166 ± 10	4 ± 1	50 ± 3	257 ± 11

## Data Availability

The data and analysis in this study are available on request from the corresponding author.
